# Steering, not drifting — a fresh approach for navigating private sector engagement for Universal Health Coverage

**DOI:** 10.1136/bmjgh-2023-014460

**Published:** 2023-12-15

**Authors:** David Clarke

**Affiliations:** World Health Organization, Geneva, Switzerland

**Keywords:** Health policies and all other topics, Health systems, Health policy

## Introduction

This Supplement delves into a long-neglected area of health system strengthening: private sector engagement. The WHO defines private sector engagement as the meaningful inclusion of private providers for service delivery in mixed health systems, emphasising the government’s need to govern the whole health system—private and public. This ensures quality of care and financial protection for patients, irrespective of where they seek care. It requires that the private sector aligns with public sector health goals and commits to supporting the government’s agenda.

Addressing the topic of private sector engagement is critical, as the private sector is an essential player in most of the world’s health systems. WHO’s figures on the sources of care across the WHO regions show that the private sector provides up to 40% of all health services in the WHO Americas region, Africa region and Western Pacific region, up to 57% of the Southeast Asia region and up to 62% in the Eastern Mediterranean region.^1^

In this context, establishing effective governance arrangements between the government and the private sector is not optional and is essential. Without inclusive and necessary policy frameworks, comprehensive data for informed decision-making, and the ability to deploy incentives, regulatory instruments and other governance tools, health systems are left to drift rather than be steered.^2^ As a result, universal health coverage (UHC) policy failure is almost inevitable, leading to the non-achievement of UHC goals of equity, efficiency and people-centred care.

## A call for a fresh approach

In 2020, Dr Peter Salama, former Executive Director of Universal Health Coverage at WHO, issued a call to action to reframe the private sector’s contribution to UHC as ‘a partnership in health for shared health outcomes’.

Recognising the need to refresh and update the stewardship concept from the World Health Report 2000, the call to action aimed to build consensus around the means and strategies for engaging the private sector in healthcare service delivery for UHC as part of the Sustainable Development Goals (SDGs) agenda.

The formal mandate for this work comes from the 63rd World Health Assembly, which adopted a resolution to engage the private sector in providing essential health services.^3^ Notable progress in implementing this resolution has been made in key priority programme areas such as tuberculosis, malaria, and maternal and child health. Since 2019, the WHO Systems Governance and Stewardship team, now part of the Primary Healthcare Special Programme, has been promoting an evolution of this work involving a system-wide shift to catalyse action for UHC, to strengthen governance of mixed health systems and assure alignment of the private sector, to promote equity, access, quality and financial protection for the population.

The desire for a new approach led WHO to establish the Technical Advisory Group on the Governance of the Private Sector for Universal Health Coverage (the TAG) and commission a Strategy Report called ‘Engaging the Private Health Service Delivery Sector through Governance in Mixed Health Systems’.^4^

## The theory of change for the new approach

The Strategy Report introduced a theory of change for new ways of doing governance, envisioning a system that aligns the heterogeneous private sector service delivery to public sector service delivery. Six key governance behaviours drive this theory of change focused on performing the practice of governance.

This core tenant of the strategy is based on the principle that a well-governed health system is a system where public and private actors collectively deliver on the realisation of UHC. The TAG recommended the performance of these governance behaviours, discussed in depth in this Supplement, to create an inclusive whole health system governance operating framework.

The purpose of an inclusive operating framework is twofold. First, to establish institutional foundations, data, and governance tools for implementing a political commitment to the principles and values of UHC, ensuring equitable access to healthcare, maintaining and improving service quality, and providing financial protection. Second, to foster partnerships for sustainable development, leveraging the resources and expertise of all health system actors, including the private sector, to address a country’s existing and emergent health challenges.

This fresh approach requires governments to embrace a new role to ‘steer’ rather than ‘row’ the health system,^5^ focusing not just on managing public services but also on guiding mixed health systems.

Implementing this shift demands a different perspective on health systems governance. While acknowledging the importance of theoretical grounding, our fresh approach views governance as a set of processes, mechanisms and tools that collectively determine how authority is exercised in a particular context. Governance is filtered through relationships, underpinned by values and norms, influenced by organisational structures and resources, and embedded in historical and sociopolitical systems.^6^

This Supplement, commissioned by WHO and *BMJ Global Health*, aims to discuss and provide evidence on private sector engagement, inclusive public policy and the practice of governance, collectively the ‘means’ to achieve UHC policy’s ‘end’ goals.

Practice has become critical for understanding central questions about how agency, structure, individual action and institutions are linked in social systems, cultures and organisations. Focusing on practice will help countries transition from theoretical understandings of governance to integrating governance tools and processes into health policy and programme design.^7^

This practice approach forms the centrepiece of the WHO strategy report’s six governance behaviours. The governance behaviours break down what have tended to be long lists of governance activities, that is, ‘ensuring [that] strategic policy frameworks exist and are combined with effective oversight, coalition-building, regulation, attention to system design and accountability’.^8^

## Conclusion

At a recent TAG meeting, Dr Suraya Dalil, Director of the WHO’s Special Programme on Primary Healthcare, issued a new call to action on private sector engagement. Dr Dalil highlighted the significant proportion of primary care services delivered by the private sector in health and emphasised the importance of private sector engagement as part of WHO’s work on PHC reform, prioritising equity, human rights and social justice.

This Supplement responds to the World Health Assembly (WHA) resolution, calling for WHO support for private sector engagement and aligns with Dr Salama and Dr Dalil’s collective call to action. In this Supplement, we aim to highlight the importance of WHO’s work programme on private sector engagement for UHC and advocate for a fresh approach to ensure that governments have the tools and knowledge needed for private sector engagement. In this way, they can be effective guarantors that health services are available to all based on need, acceptable and of good quality wherever people can access them.

## Data Availability

Data is avaialble in a publci access repositry.
